# 

**Published:** 1876-09

**Authors:** 


*CONTENTS.*

| | PAGE |
|---|---|
| WAKEFULNESS | 373 |
| LATE SUPPERS | 375 |
| WEARING GLASSES | 377 |
| A RANK OFFENCE | 379 |
| BOWEL COMPLAINTS OF CHILDREN | 380 |
| VARIETY IN DIET | 382 |
| THE HEALTH FOOD COMPANY | 386 |
| SARATOGA SPRINGS | 390 |
| COOKED MEATS | 393 |
| A QUESTION OF FOOD | 394 |
| MASKS | 396 |
| PERTAINING TO INSURANCE | 397 |
| MALARIA | 399 |
| SIMILARITY OF INDIVIDUALS AND SOCIAL ORGANISMS | 400 |
| ROSERL | 401 |

